# Newly diagnosed B-cell acute lymphoblastic leukemia demonstrating localized bone marrow infiltration exclusively in the lower extremities

**DOI:** 10.1515/med-2025-1368

**Published:** 2025-12-30

**Authors:** Yaqiu Wang, Peng Sun, Shunxia Hu, Yanzhen Wan

**Affiliations:** Clinical Laboratory, Women and Children’s Hospital, Qingdao University, Qingdao, China

**Keywords:** acute lymphoblastic leukemia, localized leukemia, differential diagnosis, musculoskeletal involvement

## Abstract

**Objectives:**

Acute lymphoblastic leukemia (ALL) is generally thought to be widely distributed in the bone marrow (BM). Localized involvement of BM from particular bones is extremely rare, especially in newly diagnosed cases. Here, we described a newly diagnosed case of B-cell ALL localized to the lower extremities in a five-year-old boy.

**Case presentation:**

The patient presented with pain in both knees for over one month. Imaging findings indicated pathological fracture of the right distal femur and bilateral proximal tibiae. An open biopsy of the right distal femur lesion was performed, and BM cytology and histopathological analysis confirmed the diagnosis of B-cell ALL. However, his whole blood and BM findings on usual biopsy sites (the sternum and iliac bone) were normal. He received standard treatments for ALL, and achieved complete remission. He has been on maintenance therapy till now without evidence of relapse for two and a half years.

**Conclusions:**

The present case highlights the fact that, in exceptionally rare circumstances, ALL may initially manifest as localized infiltration within BM of specific bones, rather than exhibit diffuse BM involvement, which renders BM aspiration and biopsy only from the usual sites insufficient for the diagnosis of leukemia. Therefore, clinicians should maintain a high index of suspicion for acute leukemia when facing any child with unexplained persistent skeletal pain and radiographic abnormalities even in the absence of abnormal hematologic findings, and strongly consider performing targeted BM biopsies of radiologically abnormal bone lesions alongside conventional BM sampling sites.

## Introduction

Acute lymphoblastic leukemia (ALL) is the most common pediatric malignancy, accounting for about one-third of all childhood cancers [1]. ALL is characterized by malignant proliferation of immature lymphoid cells, which can invade bone marrow (BM), blood, and extramedullary sites such as musculoskeletal system, kidney, liver, the central nervous system, and testicles, etc. [2]. Initial manifestations prior to diagnosis are usually non-specific, mimicking those common self-limiting diseases, which make timely and accurate diagnosis more challenging and difficult.

The most common symptoms of acute leukemia in children include hepatomegaly, splenomegaly, pallor, fever, and lymphadenopathy [3], 4]. Musculoskeletal involvement has also been reported to be frequently observed in children with leukemia, presenting with limb pain, bone pain, musculoskeletal impairment, joint pain, and limp [4]. ALL is generally thought to be widely distributed in the BM. Localized involvement of the BM of a particular bone is extremely rare, especially in newly diagnosed cases. In the present study, we reported a case of B-cell ALL exclusively localized to the lower extremities without abnormal BM findings on the usual biopsy sites in a five-year-old boy.

## Case presentation

**Figure 1:**
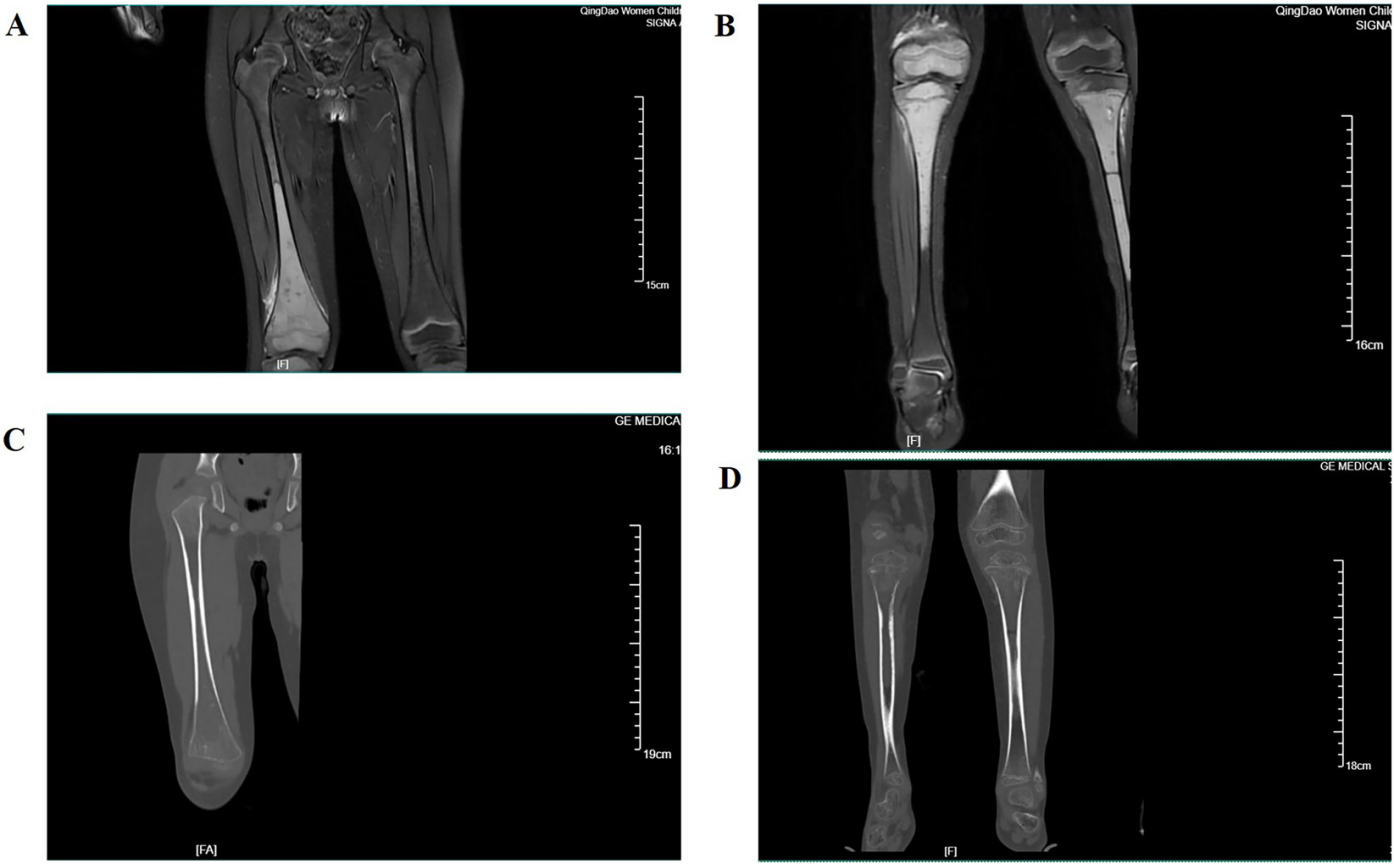
Findings of MRI and CT scan revealed pathological fracture of right distal femur and bilateral proximal tibiae, and indicated the possibility of metabolic bone disease or tumor-like lesion. A. MRI scan of bilateral femurs. B. MRI scan of bilateral tibiae and fibulae. C. CT scan of right femur. D. CT scan of bilateral tibiae and fibulae.

A five-year-old boy was admitted to the Orthopedics Department of Qingdao Women and Children’s Hospital complaining of pain in both knees, leading to his refusal to walk for more than one month without obvious swelling and skin damage. He had no medical or family history of genetic disorders. He had a fever lasting for 2–3 days with a peak temperature of 38.3 °C, which returned to normal after taking oral antipyretic drugs. After admission, physical examination revealed tenderness in the right distal femur with slight restriction of movement, no movement restriction of the left knee joint and hip joint, no other obvious abnormalities of the limbs, no enlargement of lymph nodes, and no palpable liver or spleen. Laboratory investigations demonstrated normal results of whole blood count with white blood cell (WBC) count 7.93 × 10^9^/L (normal reference range, 4.9–12.7), normal blood film, slightly increased erythrocyte sedimentation rate 20 mm/h (normal reference range, 0–15), normal levels of tumor biomarkers (AFP, CEA, CA199, CA153, Ferritin), normal calcium and parathyroid hormone levels, decreased 25-OH-vitamin D level 12.9 ng/mL (normal reference range, >20 ng/mL), and basically normal levels of biochemistry parameters (with lactate dehydrogenase (LDH) slightly increased (317.8 U/L, normal reference range 120–250 U/L)). On the admission day, digital radiography (DR) showed osteoporosis of both knee joints and multiple low-density shadows of saccular or cribriform shapes in the right distal femur. Magnetic resonance imaging (MRI) revealed a slightly enlarged metaphysis of the right femur compared to the left side, intramedullary abnormal signal intensity in the right middle and distal femur, epiphysis accompanied by local pathological fracture, slightly enlarged metaphysis of bilateral tibiae, intramedullary abnormal signal intensity in the bilateral proximal and middle tibiae, epiphysis of the right tibia, and metaphysis of the bilateral fibulae (shown in Figure 1a and b). On day 2, computed tomography (CT) images showed slight enlargement of the right distal femur and bilateral proximal tibiae, bone discontinuity, and local high-density shadow of bone trabeculae in the metaphysis of the right distal femur and bilateral proximal tibiae, and decreased bone density, sparse bone trabecula, and inhomogeneity of medullary cavity density in the distal femur, bilateral proximal and middle tibiae, and metaphysis of the right proximal fibula (shown in Figure 1c and d). Imaging findings indicated pathological fracture of the right distal femur and bilateral proximal tibiae, and the possibility of metabolic bone disease or tumor-like lesions.

**Figure 2:**
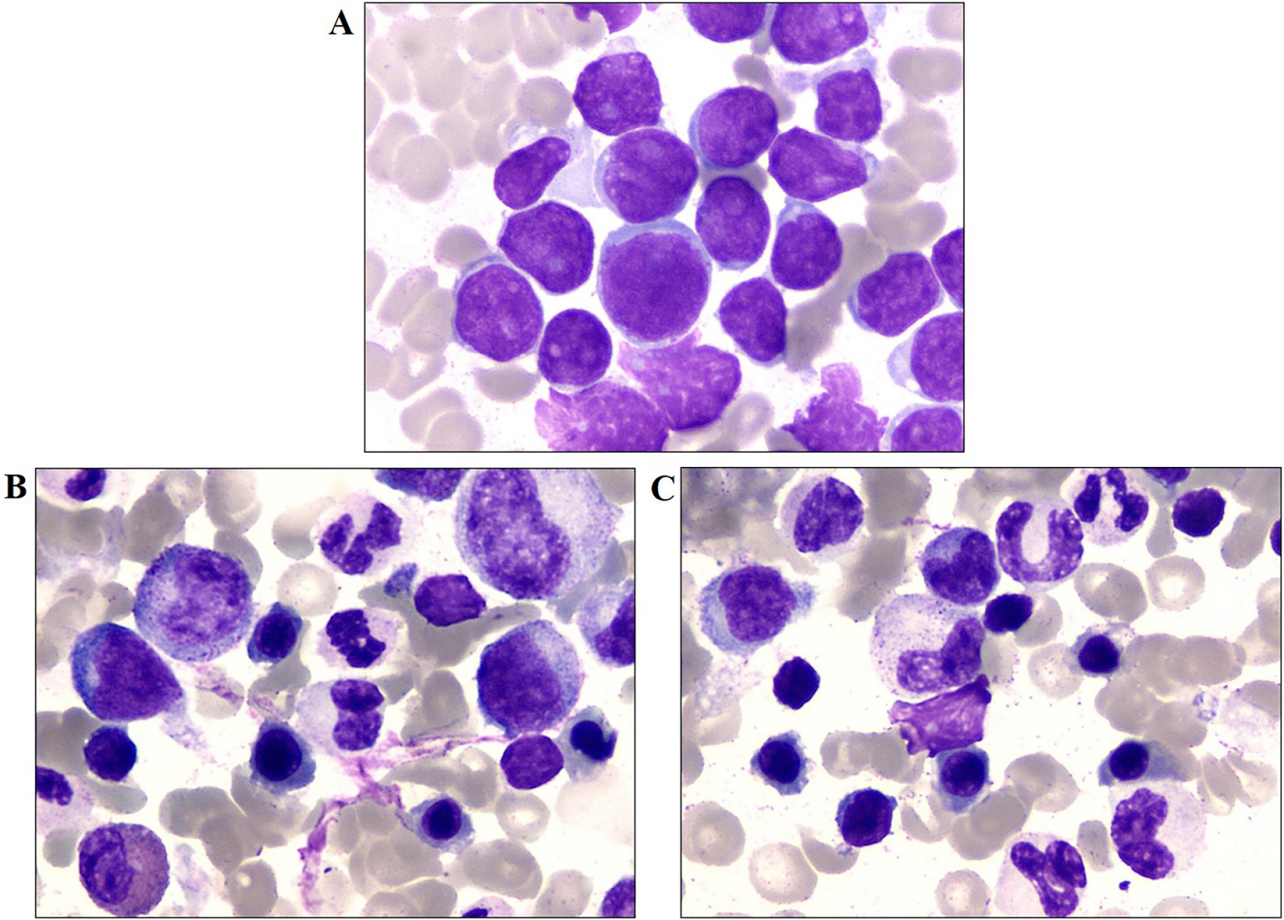
Bone marrow cell smear ( × 100 objective; wright–giemsa staining). A. Bone marrow from right femur (92.0 % lymphoblasts). B. Bone marrow from the sternum (generally normal trilineage hematopoiesis). C. Bone marrow from the right iliac bone (generally normal trilineage hematopoiesis).

**Figure 3:**
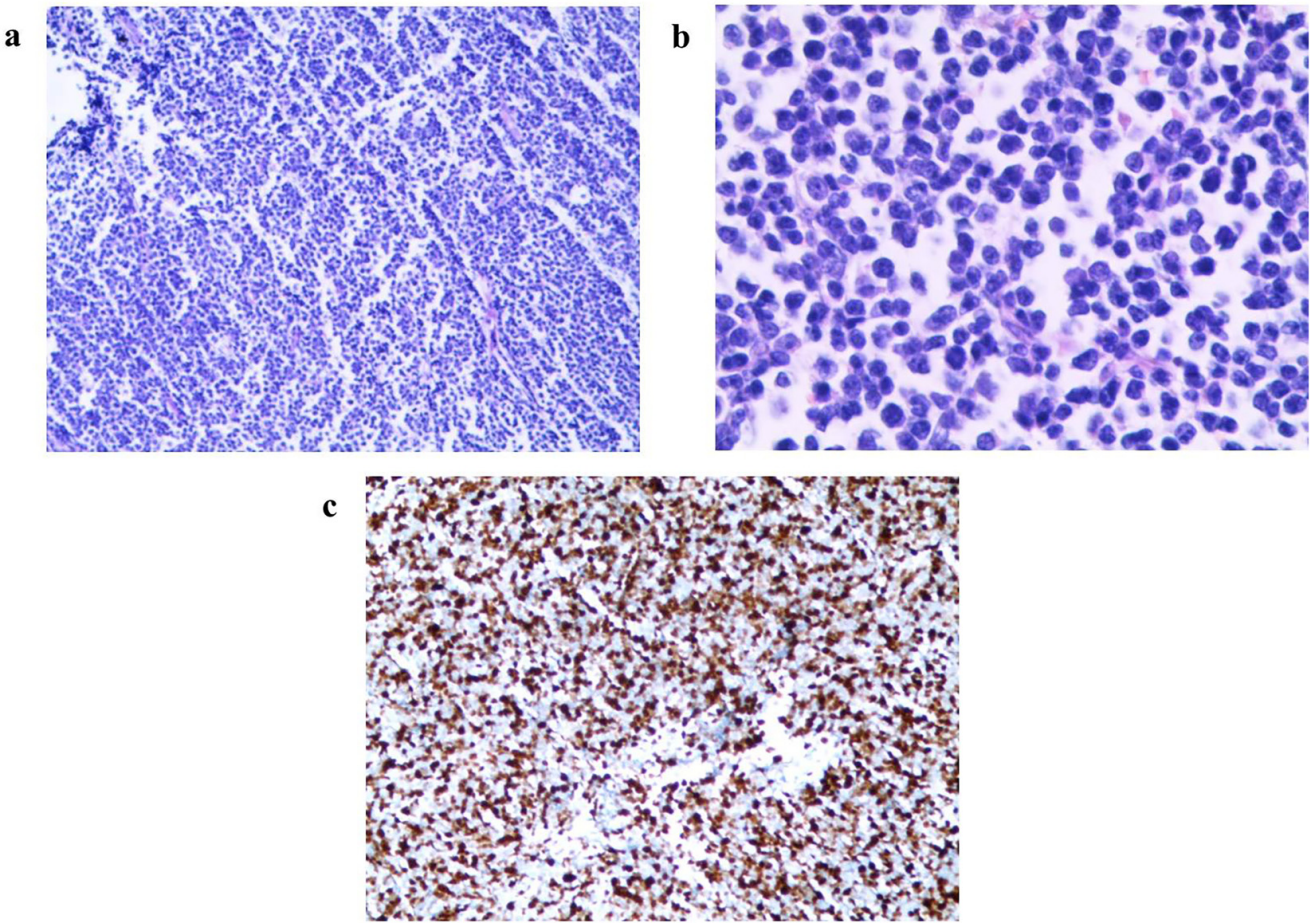
Histopathological analysis and immunohistochemical staining of the biopsy tissue from the right distal femur lesion. A-B. Histopathological analysis of the biopsy tissue. (Hematoxylin and eosin stain, a. × 10 objective, b. × 40 objective). C. Immunohistochemical staining of Ki67 ( × 10 objective).

To confirm the diagnosis, three days after admission, an open biopsy of the right distal femur lesion was performed under general anesthesia for histopathological examination, and BM was aspirated for culture and cytology analysis. BM cytology analysis revealed 92.0 % lymphoblasts in the right distal femur, indicating the possibility of ALL (shown in Figure 2a). Histopathological analysis indicated high density of primitive cells with morphological uniform, which presented diffuse infiltration, and no normal bone marrow structure (shown in Figure 3a and b). Immunohistochemical staining showed that the tumor cells were CKpan -, Vim+, TdT +, CD20 -, CD79α +, CD3 -, CD43 +, NSE -, Syn -, CgA -, CD99 +, FLi1 +, Myogenin -, MyoD1 -, and part of the tumor cells were positive for LCA. The positive rate of Ki67 was 70 % (shown in Figure 3c). Pathology consultation confirmed the diagnosis of B-cell ALL. The patient was then transferred to the Hematology Department, and the sequential chemotherapy for ALL was initiated four days after admission. To evaluate risk classification and complete immunophenotyping, molecular subtyping, and chromosome analysis, BM aspiration from the sternum was performed on day 5. However, cytological analysis revealed normal trilineage hematopoiesis in the sternum (shown in Figure 2b), and flow cytometry showed no evidence of acute leukemia, non-Hodgkin’s lymphoma, or high-risk myelodysplastic syndrome. Chromosomal analysis results were normal (46, XY). Gene mutation screening for hematological diseases revealed no hotspot mutants, whereas possible disease-associated mutant sites were found in BCL6 (49.1 %) and FAT1 (50 %). The quantification of EVI 1 was 0.34, and KMT2A, PDGFRB, MYC, ETV6:RUNX1, and BCR::ABL156 were all negative. In addition, 56 fused genes associated with leukemia were negative. On day 9, BM aspiration from the right iliac bone was performed, and BM cytology analysis revealed no obvious abnormalities (shown in Figure 2c).

Eventually, the boy was diagnosed with B-cell ALL localized to the lower extremities, mainly involving the bones and BM around the knee joints. He received standard treatments for ALL, consisting of the VDLD regimen, the CAM regimen, the mM regimen, the VDLD plus CAM regimen, and the 6-MP plus MTX regimen. The patient achieved complete remission with BM minimal residual disease (MRD)<0.01 %. He followed medical advice well, and has been on maintenance therapy with vindesine plus dexamethasone under the care of pediatric hematologists for 10 cycles without evidence of relapse for two and a half years.

**Statement of Ethics:** Written informed consent was obtained from the patient’s legal guardian for publication of this case report and any accompanying images.

## Discussion and conclusions

ALL, as the most common childhood malignancy, is a lymphoproliferative disorder. It is universally accepted that this disease generally affects the whole BM instead of some particular bones, especially in new cases. In the present study, we described a newly diagnosed case of B-cell ALL exclusively localized to the lower extremities, with normal BM findings from the sternum and iliac bone.

The clinical symptoms of acute leukemia are secondary to the inhibition of normal hematopoiesis and malignant proliferation of lymphoblasts, which mainly include asthenia, pallor, fever, hepatosplenomegaly, lymphadenopathy, and bleeding. Musculoskeletal system manifestations, such as limb pain, joint pain, musculoskeletal impairment, and limp, also feature eminently. A meta-analysis of the clinical presentation of childhood leukemia showed that 43 % of children with leukemia presented with limb pain [4]. In a study involving 122 children with acute leukemia, Sinigaglia et al. found that 38.3 % of the patients had complaints related to the musculoskeletal system at their first medical consultation with various and nonuniform localizations, and 40.2 % showed one or more radiographic abnormalities at the onset of the disease [5]. Another study demonstrated that 22 % of the 328 children affected with acute leukemia had clinical presentations associated with the musculoskeletal system at diagnosis [6]. In addition, a retrospective study involving 765 children with ALL showed that 31.4 % of the children presented with musculoskeletal manifestations (MSMs). Furthermore, MSMs were more frequently observed in B-cell ALL than in T-cell ALL, and patients with MSMs showed a lower incidence of abnormal hematological indices at diagnosis, such as lower white blood cell count and lower percentage of lymphoblasts in the peripheral blood, and less common hepatomegaly and splenomegaly than those without MSMs [7]. Remarkably, in some cases of ALL, MSMs were the only initial symptoms that can mimic orthopedic disorders, leading to misdiagnosis or delayed diagnosis, and such patients were frequently referred to the orthopedic clinic [8], 9]. A case of a femoral diaphyseal stress fracture as the initial presentation of acute leukemia in a 14-year-old boy was reported in 2016 [10]. Our patient was also a case with complaints of knee pain and refusal to walk as the only initial presentation and normal complete blood count at admission. Considering that ALL is the most frequent childhood malignancy that presents with musculoskeletal symptoms, excluding musculoskeletal tumors, it is imperative that ALL should be considered for differential diagnosis when facing any child with unexplained persistent skeletal pain or radiographic abnormalities.

ALL localized to the BM of specific bones is extremely rare and the mechanisms involved remain unclear. Few cases of focal BM involvement have been reported, mainly in relapsed ALL. In 2004, Endo T et al. described the first case of localized relapse of ALL in BM of extremities in a 37-year-old woman after allogeneic stem cell transplantation, with no morphological findings of relapse in BM from the sternum and right iliac crest [11]. Childhood ALL relapse was shown in a 28-year-old man with localized ankle pain and no abnormal BM findings on usual biopsy sites [12]. For newly-diagnosed ALL, to the best of our knowledge, there is only one similar case reported up to now. A 9-year-old girl presenting with periodic fever and left hip pain was ultimately diagnosed with B-cell ALL localized to the left pelvic BM with 99 % lymphoblasts in the left iliac BM while normal trilineage hematopoiesis was observed in the right iliac BM [13]. And she obtained complete remission with standard treatment and has been maintained for 6 years. Our patient was another case of localized B-cell ALL to the BM of particular bones. In contrast, in our case, leukemic cells were localized to the BM of the bones around the knee joints, and no abnormal findings were observed in BM aspirates from the sternum and iliac bone, which indicates that BM aspiration and biopsy from these commonly used sites are not always sufficient for the diagnosis and evaluation of acute leukemia. Under such rare conditions, magnetic resonance imaging (MRI) and fluorodeoxyglucose position emission tomography (FDG-PET) are useful for auxiliary diagnosis. Case reports have demonstrated the diagnostic value of FDG-PET in various types of leukemia. FDG-PET is a reliable, non-invasive imaging tool for the diagnosis and staging of hematological malignancies. A review of 18F-FDG-PET results showed that multiple bone foci or systemic BM uptake was observed in all 23 cases of ALL [14]. For our patient, MRI and CT findings revealed pathological fracture of right distal femur and bilateral proximal tibiae, and indicated the possibility of metabolic bone disease or tumor-like lesions. Subsequently, an open biopsy of the right distal femur lesion was performed, and the final diagnosis of B-cell ALL was confirmed. As for treatments, although leukemic cells were localized at diagnosis, systemic dissemination might be a subsequently occurring event. Therefore, the patient received standard therapeutic regimens for B-cell ALL and achieved complete remission.

This is an extraordinarily exceptional case of a newly diagnosed B-cell ALL exclusively localized to the lower extremities. The present case highlights the fact that, in exceptionally rare circumstances, ALL may initially manifest as localized infiltration within BM of specific bones, rather than exhibit diffuse BM involvement. In this particular situation, BM aspiration only from the usual biopsy sites are not sufficient for the diagnosis of leukemia. Therefore, clinicians should maintain a high index of suspicion for acute leukemia when facing any child with unexplained persistent skeletal pain and radiographic abnormalities even in the absence of abnormal hematologic findings, and strongly consider performing targeted BM biopsies of radiologically abnormal bone lesions alongside conventional BM sampling sites.
